# MOX Nanosensors to Detect Colorectal Cancer Relapses from Patient’s Blood at Three Years Follow-Up, and Gender Correlation

**DOI:** 10.3390/bios15010056

**Published:** 2025-01-16

**Authors:** Michele Astolfi, Giulia Zonta, Cesare Malagù, Gabriele Anania, Giorgio Rispoli

**Affiliations:** 1 Department of Neuroscience and Rehabilitation, University of Ferrara, 44121 Ferrara, Italy; michele.astolfi@unife.it; 2 SCENT S.r.l., Via Quadrifoglio 11, 44124 Ferrara, Italy; giulia.zonta@unife.it (G.Z.); malagu@fe.infn.it (C.M.); 3 Department of Physics and Earth Science, University of Ferrara, 44121 Ferrara, Italy; 4 Department of Medical Sciences, University of Ferrara, 44121 Ferrara, Italy; ang@unife.it

**Keywords:** chemoresistivity, gas sensor, nanotechnology, VOCs, blood, colorectal cancer, follow-up, preventive screening

## Abstract

Colorectal cancer represents 10% of all the annual tumors diagnosed worldwide, being often not timely diagnosed, because its symptoms are typically lacking or very mild. Therefore, it is crucial to develop and validate innovative low-invasive techniques to detect it before becoming intractable. To this aim, a device equipped with nanostructured gas sensors has been employed to detect the airborne molecules of blood samples collected from healthy subjects, and from colorectal cancer affected patients at different stages of their pre- and post-surgery therapeutic path. Data was scrutinized by using statistical standard techniques to highlight their statistical differences, and through principal component analysis and support vector machine to classify them. The device was able to readily distinguish between the pre-surgery blood samples (i.e., taken when the patient had cancer), and the ones up to three years post-surgery (i.e., following the tumor removal) or the ones from healthy subjects. Finally, the correlation of the sensor responses with the patient/healthy subject’s gender was investigated, resulting negligible. These results pave the path toward a clinical validation of this device to monitor the patient’s health status by detecting possible relapses, to parallel to clinical follow-up protocols.

## 1. Introduction

**Figure 1:**
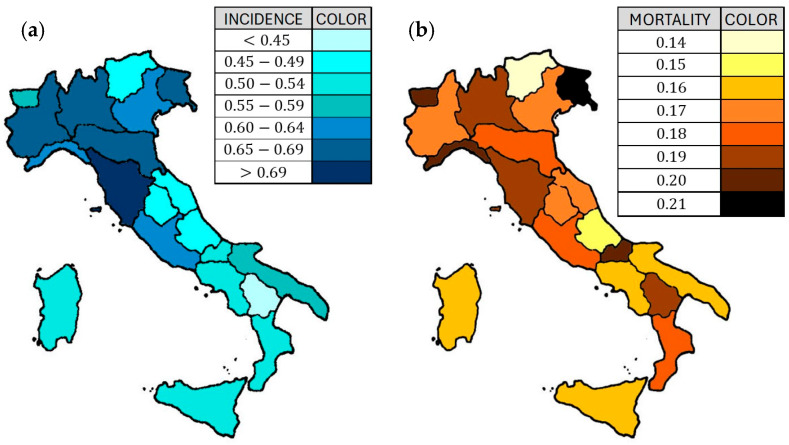
Map of the CRC incidence (**a**) and death (**b**) percentages in the Italian regions [6].

The 2024 cancer statistics underline some critical trends in cancer incidence and mortality, expecting more than 20 million new cases worldwide, and the prevision foresees its increase to 30 million new cases within 2040 [1]. The most diagnosed cancers are prostate, lung, colorectal, and stomach ones in men, while breast, lung, colorectal, and cervical ones in women, and these eight cancer types account for nearly half of all new diagnoses [1]. The predicted number of deaths due to cancer disease is about 11 million in 2024, especially in the less developed countries where sanitary conditions are very poor and medicines and treatments are not cutting-edge, so significantly reducing the patient’s survivor probability. In particular, the colorectal cancer (CRC) is ranked as the second most diagnosed tumor in women (after breast cancer) and the third in men (after lung and prostate cancers), representing about the 10% of all the annual tumors diagnosis worldwide (8–9% in USA) [1,2]. In Italy, the CRC rate of incidence is about 12–13% and more than 50,000 new cases are diagnosed per year, making it the second most diagnosed cancer for both male and female populations, though the survivor probability, currently about the 65–70%, is improving year by year thanks to advanced early screening and medical/surgical treatments [3]. The CRC symptoms could often be very mild or completely absent, leading to a late clinical diagnosis; for this reason, it is crucial to perform effective early screening to identify neoplastic formations before their degeneration and/or metastasis. Italy and other developed countries, as USA, UK, France, Australia, etc., are using the fecal occult blood test (FOBT) as preventive screening for CRC in the population at risk [4]. Whenever the FOBT is positive, the colonoscopy is strongly recommended, despite its discomfort, invasiveness and dangerousness for the patient (it could indeed cause parietal lesion or even perforation) [5]. In any case, the colonoscopy is currently considered as the gold-standard for the CRC diagnosis, because it allows a direct visualization of the colic mucous, possible biopsy collection from suspect formations, and the complete removal of small polyps. As an example, even in a territory as small as Italy, there are differences among the various Italian regions on CRC incidence and mortality [3,6,7]. These differences arise by the diverse regional pollution level, diet type, population age, and lifestyle, but also by the patient propensity to adhere to screening campaigns, the access care, the physician competence, and the progress of the cures and hospital equipment, that stem from the average educational and wealth level of the population (Table 1 and Figure 1). The most industrialized regions such as Lombardy, Veneto and Emilia-Romagna have a higher absolute number of cancer deaths, but in percentage terms their mortality is in line with the national average. The southern regions (such as Campania, Apulia, Calabria) and the islands (Sicily, Sardinia) have similar mortality rates, but some areas in the south show a slightly higher percentage due to various factors, including access to care and early diagnosis. Liguria and Friuli Venezia Giulia have higher mortality rates than the national average, also due to an older population [6].

The five-year follow-up program undertaken from patients after the surgical/pharmaceutical treatment is crucial to monitor the patient’s health status and to detect possible relapses [8]. The follow-up steps include various expensive and invasive analysis techniques: colonoscopy, usually performed every 1–3 years to check for any new polyps or neoplastic formations in the colon and rectum; abdomen computed tomography and/or magnetic resonance, to image any possible sign of recurrence, especially if the disease stage was advanced; blood tests, as the carcinoembryonic antigen test, useful to detect possible cancer relapse or progression [9,10]. These steps are officially recommended by the American Cancer Society and by the guidelines of the Associazione Italiana Oncologia Medica (CRC 2024 AIOM) [4]. On this basis, the research for effective and non-invasive CRC screening techniques is crucial to reduce patient risks and to improve the probabilities of detecting the disease in early stage. Among them, the Fecal DNA test is surely a novel and promising (but very expensive) technique for CRC screening, showing encouraging sensitivity and specificity values of 92% and 87% respectively for III and IV tumor stage, but they sensibly decrease for I and II tumor ones [11,12]. Therefore, the research of innovative and non-invasive methods to accurately detect specific CRC liquid and/or gaseous biomarkers is currently broadly pursued. A large number of studies demonstrated that cancer cells discharge in the blood stream specific organic compounds strictly related with their altered metabolism occurring during the carcinogenesis, i.e., the process for which normal cells are transformed into cancer ones, including several genetic and molecular changes that lead to uncontrolled cell proliferation and growth [13,14,15]. Moreover, tumor cells force the formation of new blood vessels (angiogenesis) in order to provide more oxygen and nutrients needed for their growth. Some of these organic compounds, having a high vapor-pressure, are volatile (volatile organic compounds, VOCs). Since these VOCs are generated by several carcinogenesis processes, as cellular membrane peroxidation [16,17], an accelerated glycolysis activity [18,19,20], etc., they could be considered as promising tumoral markers. The VOCs detection could have a huge potential in the clinical practice because it could be performed as screening and follow up in-vitro techniques for tumor prevention and monitoring [14,21,22,23,24,25,26,27,28,29,30]. In the last decade, some attempts were done to quantitatively and qualitatively study the cancer VOCs, mainly employing gas-chromatography-mass spectrometry (GC-MS), which identified with a good likelihood different VOCs related to cancer diseases [27,31,32,33,34,35,36,37,38]. Although the detection power of GC-MS, it presents several disadvantages: it consists in a very expensive and bulky setup that needs costly maintenance and highly trained personnel; its operation foresees the use of expensive gases, solvents, and consumables; its outcomes are often hardly to interpret; it foresees long and complex data acquisition and analysis. For these reasons, GC-MS results are suitable for research purposes (used so far for breath analysis) but not for clinical practice. Given that, the chemoresistive nanostructured gas sensors, based on metal-oxide/sulfide semiconductor materials, could be a viable alternative for the non-invasive VOCs analysis. The sensors employed in this work are entirely realized at the Sensor Laboratory (SL) of the Department of Physics and Earth Sciences of the University of Ferrara (UNIFE). Given their low cost, ease to use, adaptability, and sensitivity, they are optimal candidates to be employed in medical devices for tumoral screening and monitoring (in this case CRC) [39,40,41]. This kind of sensor, despite their poor selectivity, demonstrated to be suitable to detect VOCs pattern variations at extremely low concentrations (up to tens part per billion), in different human fluids and samples such as feces, blood, intestine biopsies, etc., as well-documented in literature. The device employed here, SCENT B2, is a new version of two patented devices, SCENT A1 and SCENT B1 (manufactured by the company SCENT S.r.l. (Ferrara, Italy). within a collaboration with SL, www.scent-srl.it, accessed on 13 January 2025), where the electronics and the dedicated software were completely updated and optimized. SCENT devices were successfully employed so far to highlight the variations of VOCs patterns occurring in human fluid samples collected from CRC-affected patients and the healthy control ones [21,26,30,42,43,44]. In particular, previous feasibility studies conducted by using SCENT B1 were able to discriminate the blood samples collected from CRC affected patients and the ones collected from presumed healthy subjects [21] and to successfully monitor the patient’s health status up to one-year follow-up [44]. Given these promising results, the purpose of this study is to employ SCENT B2 to perform a three-year follow-up on CRC patients surgically treated and to perform a gender correlation. Indeed, the incidence of most of the nonreproductive tumors in females is lower than in males, being the mortality rates of the former about one half of the latter. Moreover, CRC-affected women 18–44 years old have a better survival outcome compared to men of the same age or compared to women over 50 years old. There are gender-related differences in the CRC anatomic location and morphology, with higher rates of right-sided colon cancers in women (which are more often flat in shape, therefore more difficult to discriminate from the surrounding tissue by colonoscopy) and higher rates of rectal cancers in men [45,46]. Therefore, gender dependency about tumor formations and treatment effectiveness is nowadays a very popular topic for the research community worldwide [47,48,49,50,51,52], hence it would be interesting to analyze all the outcomes obtained in our studies carried out so far sex differences [21,30,44]. Furthermore, the ability of the intestinal epithelial cells to metabolize sex steroids, particularly estrogens, could influence the development of CRC: this would justify the estrogens protection against CRC progression and the increase of CRC risk by androgens. The gut microbiota may influence CRC development through different mechanisms (as the undigested fiber fermentation, regulation of the host immune system, and production of carcinogenic compounds) and sex hormones regulate its composition and function and its metabolites [53]. Tumors are characterized by an aberrant metabolism, aimed to increase the energy production and the macromolecules synthesis for cell growth that appears to be sex-specific. For CRC affected women, tumoral cells uses preferentially oxidation of fatty acids cells to generate the energy necessary to promote its growth, whereas in men they have an increased glycolysis [54]. This might lead to a different VOCs pattern, that could be identified by the device presented here.

## 2. Materials and Methods

### 2.1. Recruited Patients

This observational, single center, and prospective study, was approved by the Ethics Committee on 13 July 2017 at the Surgery Department of “Azienda Ospedaliero-Universitaria di Sant’Anna di Cona (Ferrara)” with number 170484. Thirty CRC-affected patients were recruited in the period between October 2020 and May 2022 at the S. Anna Hospital of Ferrara (after signing informed consent for clinical data collection) and they were followed up to June 2024 to monitor their healing status by measuring their blood VOCs. The patients had an average age of 69, and their clinical data are reported in Table 2.

Patients were included in this study if aged over 18 years old and had the CRC removal through laparoscopic or laparotomic elective surgery. The patients excluded were: pregnant women, subjects treated in emergency surgery, or the ones having other evident intestinal pathologies.

Blood samples were gathered from CRC patients at the Hospital S. Anna in 7 cc vials containing the anticoagulant agent K3-EDTA along their therapeutic path, as follows:

- T1: the day of the surgical treatment, but before the surgery;
- T2: the day of the patient hospital discharge (with a temporal distance from T1 depending upon the patient clinical course, but no longer than two weeks);
- T3: at least one month after T1 (asking the same patient a return to the hospital);
- T4: 10–12 months after T1 (second return to the hospital);
- T5: about three years after T1 (third return to the hospital).

### 2.2. The Device and the Sensors

**Figure 2:**
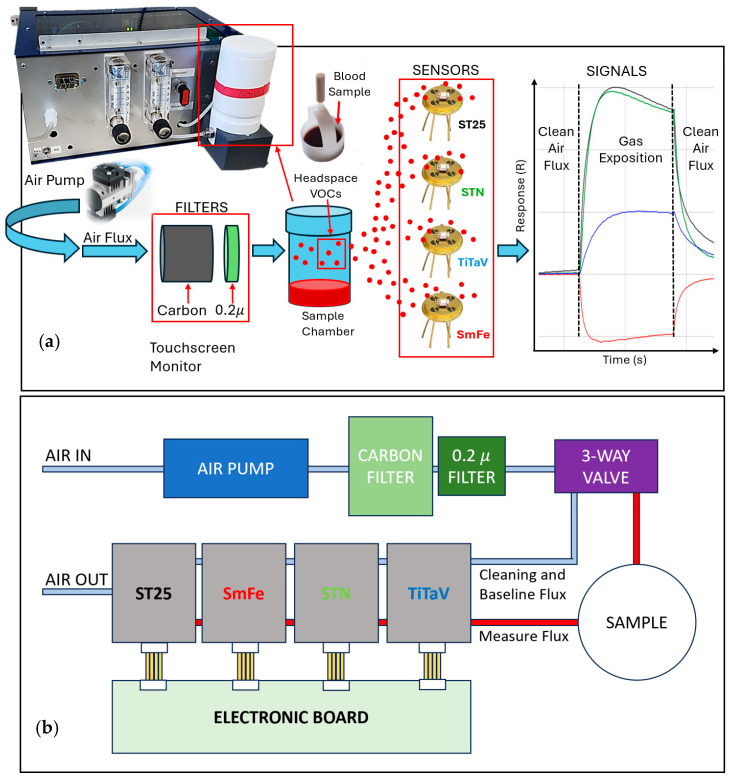
Sketch of the device and the hosted sensors. (**a**) Diagram of the device (**top left**) and how it is used: a clean and humidity-stabilized air flux is conveyed at first toward the sensors to set $V_{Air}$, and then through a box (framed by the large red rectangle) holding the blood sample to gather the headspace VOCs (small red rectangle) and finally toward the sensors, giving $V_{Gas}$. The films of the sensors hosted in the device are obtained by mixing different metal-oxide nanopowders as follows: ST25 + 1% Au (or ST25; tin and titanium with the addition of 1% of gold nanoparticles), SmFeO_3_ (samarium and iron), STN (tin, titanium, and niobium), and TiTaV (titanium, tantalum, and vanadium); the sensor responses to a blood sample of a CRC-affected patient are shown on the right; each response is identified to the corresponding senor by a dedicated colour; (**b**) Block scheme of SCENT B2 device.

SCENT B2 is a hand-made prototype, entirely developed by SCENT S.r.l. company in collaboration with the “Laboratorio Sensori” of the Department of Physics and Earth Science, University of Ferrara (Figure 2) [55]. SCENT B2 is made up of a pneumatic system, necessary to direct the sample headspace compounds toward the sensors, and an electronic one, assigned to supply the sensor heater and film, and to digitize and acquire the sensor signals (Figure 2). SCENT B2 is connected through a serial cable to an external computer (with a touchscreen monitor) which, through a dedicated software, allows to manage the sensors feedings (heater and film) and their signal magnification, to plot the signals in real-time (automatically computing the response curve as a function of time), and to acquire and store them in a .csv or .txt file as the user prefer (Figure 2a). Briefly, as schematically shown in Figure 2a,b, a micro air pump (max flow rate of 1 L/min) generates a clean (by using an homemade carbon filter and a 0.2 micron (Merck, Darmstadt, Germany) one and stable (regulated with a fluxmeter (Key Instruments, Croydon, PA, USA) to about 0.2 L/min) air flux. This flux can be rerouted by a three-way manual valve: directly to the sensors, creating a reference signal (baseline, $V_{Air}$) and/or to “wash” the sensors after a gas exposition (Figure 2b, light blue line), or first to the sample chamber, where it picks up the sample headspace gaseous compounds, and then to the sensors, where the sample gases interact with the sensor surface (Figure 2b, red line).

**Figure 3:**
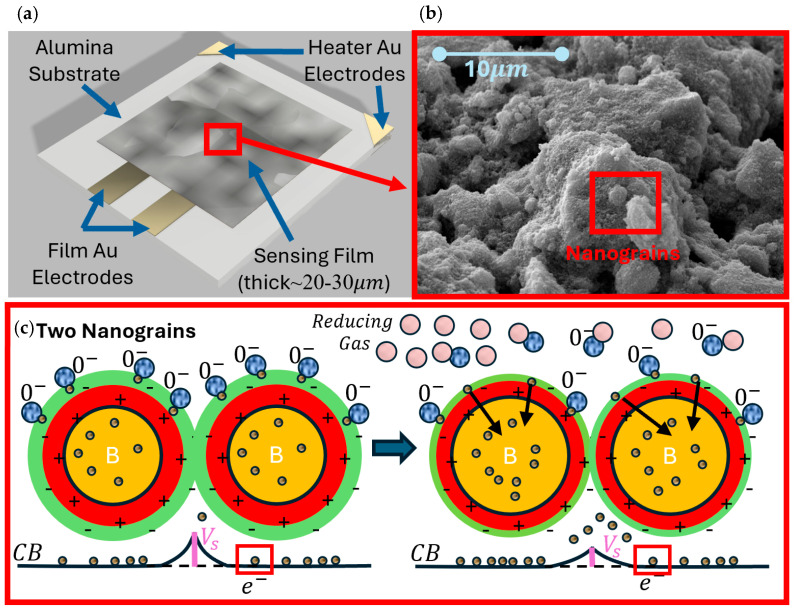
Scheme of the working principle for a n-type chemoresistive sensor. (**a**) Cartoon of the film, which is held by an alumina substrate hosting the heater (placed on its bottom side) and four gold contacts: two for the heater feeding and two for the sensing film signal; (**b**) enlargement of a portion of the STN sensor film (square red box in (**a**)) by SEM imaging; (**c**) a cartoon of a further enlargement of the square box in (**b**); the sensor grains placed in a reference atmosphere (left, dry air) interact with the oxygen species (as O^−^ or O_2_, blue circles), which entrap the bulk (orange area) electrons (small yellow circles) at the sensor grain surface (the external negative green shell), so enlarging the depletion region (positive red shell) and increasing the grain–grain potential barrier (*V_S_*). Right panel, a reducing gas (as CO, CO_2_, H^+^, etc., pink circles) is annealed on the nanograins surface where it reacts with the adsorbed oxygen, freeing electrons in the conduction band (CB), causing a *V_S_* decrease, and therefore a current increase.

The measurement process foresees the following steps: once the baseline in the reference atmosphere (in this case dry air) was achieved for all sensors, the blood stored in a 7 cc vial was poured inside an aseptic Teflon container that was placed as fast as possible in the SCENT B2 sample container (white cylinder in Figure 2), to minimize any possible VOCs loss and contamination by external air. Then the air flux was routed to reach before the sample chamber and then the sensors (Figure 2b, red line) by setting the three-way valve in the “measure” position. In this configuration, the sample headspace gaseous compounds carried by the air flux interacted with sensors film surfaces, inducing their resistance variation (signals as function of time; Figure 3). Once these signals reached a plateau ($V_{Gas}$), the air flux was conveyed again toward the sensors (by returning the three-way valve to the “rest” position), in order to recover them to the initial baseline, while the blood sample was properly disposed. The sensor response R as a function of time was computed as the ratio between the voltage at the plateau in sample gas presence ($V_{Gas}$, applied in between the two dotted vertical black lines in Figure 2a, right panel) and the one in the presence of a clean airflow, when the sensor recovery was completed ($V_{Air}$, before and after the two dotted black lines, Figure 2a, right panel):

(1) $$R=\frac{V_{Gas}}{V_{Air}}$$

R is therefore dimensionless and is independent from the measured physical quantity and the baseline amplitude (in general different for each sensor) [21,42].

SCENT B2 is equipped with the following four sensors:

- ST25 + 1% Au (or ST25): a mixture of tin and titanium oxides with the addition of 1% of gold nanoparticles (n-type);
- SmFeO_3_: samarium and iron oxides (p-type);
- STN: tin, titanium, and niobium oxides (n-type);
- TiTaV: titanium, tantalum, and vanadium oxides (n-type).

Typical sensor responses are shown in Figure 2a (ST25, black line; SmFe, red; STN, green; and TiTaV, blue) during the application of the gas exhaled by the blood sample (here from a CRC-affected patient; the application timing in between the two dotted vertical black lines is lasting about 10 min). Data were further processed by employing several statistical techniques exploiting the SciPy library of Phyton 3.10.

The working principle of the thick-film sensors employed in SCENT B2 is the chemoresistivity, consisting in a change of their sensing film resistance as a result of the chemical redox reactions occurring between their surface and the gaseous molecules of the surrounding atmosphere [56,57,58] (Figure 3). Since the electrons of the sensing film material are promoted in conduction band (CB) mainly through the termionic effect, the film must be thermally activated (at about 350–450 °C) by means of a heater (platinum meander) serigraphically printed on the sensor substrate bottom side (Figure 3).

This thermal activation maximizes the number of electrons in CB, having enough energy to overcome the grain-grain potential barrier, and, at the same time, high temperature ionizes the gaseous molecules in the atmosphere surrounding the sensor, promoting their adsorption on its surface. Therefore, the film sensor (synthesized with the sol-gel technique [59,60,61]) is heated to a suitable working temperature (WT) through a filament placed below the film, so to maximize the sensor response amplitude and repeatability [62,63].

Among the four sensors equipping SCENT B2, just the ST25 + 1% Au and STN (both heated at 450 °C) were able to successfully detect and distinguish the cancer and healthy blood exhalations, as shown in feasibility studies [21] and in a previous follow-up paper [44], so only the responses of these two sensors were considered here.

### 2.3. Sample Handling

**Figure 4:**
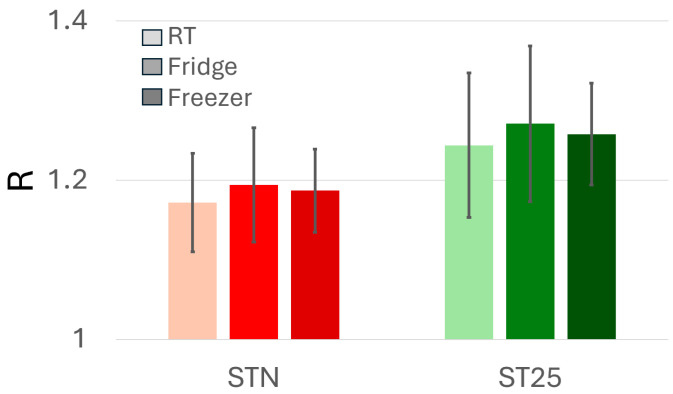
Average sensor responses (R) to the exhalations of the same blood samples stored at different temperatures. STN (red shades) and ST25 + 1%Au (green shades) responses to the same blood sample kept for one hour at room temperature (light colours), refrigerated (4 °C; dark colours) and frozen (−20 °C; darkest colours).

To find out the most suitable blood conservation, a group of six healthy patients (comprising some authors of this paper) gave 21 cc of blood that was divided in three 7 cc vials: one was stored at room temperature, one at 4 °C, and one was frozen at −20 °C. After one hour from blood collection, the frozen sample was thaw and the three samples were measured in sequence (as detailed below) by the ST25 + 1% Au and STN sensors and their responses were averaged and compared (Figure 4).

Since all these blood exhalations were almost identical, no matter if the sample was kept at room temperature, refrigerated or frozen, then the samples were stored at room temperature in K3-EDTA-cured vials and measured within one hour from collection.

### 2.4. Data Analysis

The data were stored and organized by using the Python libraries Pandas and NumPy. All the statistics (parametric and non-parametric significance tests, Levene test, Cohen d, confidence intervals, and principal component analysis (PCA)) were performed by using custom Python programs by exploiting the most suitable statistical libraries as SciPy, Stats, etc.; the support vector machine (SVM) analysis was performed by using the Sklearn library.

## 3. Results and Discussion

### 3.1. Ensemble Statistical Analysis

The blood of thirty CRC patient was collected at five different stages of their surgical clinical path (T1–T5, see Section 2), for a total of 135 blood samples (instead of 150, because some patients were not able to provide all the five samples for several reasons as early dehospitalization, death, etc.). Just after the surgery (T2) and after one month of follow up (T3) the sensor responses were paradoxically larger than the pre-surgery ones (T1), probably due to the interference of the anaesthesia exhalation, the adjuvant chemotherapeutic drug treatments, and the increased perioperative catabolism.

As anticipated above, the best sensors to detect tumoral VOCs from blood samples were ST25 and STN sensors (both based on a mixture of Tin and Titanium oxides: 75% − 25% and 70% − 30% + 1% of external addition of Niobium, respectively). They showed the highest discrimination between tumor affected and healthy samples and resulted to be the sensor materials less affected by humidity (since it generally could compromise the sensor reliability and it typically slows down the sensor recovery time) [64,65,66,67].

**Figure 5:**
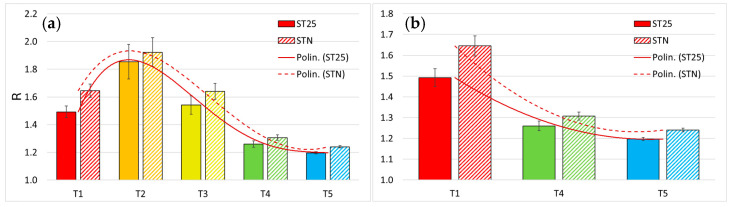
Sensor responses to the blood exhalations of patient before and after surgery. (**a**) Solid bars and striped ones are the R amplitude for ST25 + 1% Au and STN sensors, respectively to the blood exhalations of the same patients at the various stages of follow-up (T1, red, n = 30; T2, orange, n = 22; T3, lime green, n = 29; T4, green, n = 28; T5, blue, n = 26); the solid and the dashed lines are the polynomial fittings to the ST25 + 1%Au and STN bars, respectively; (**b**) Comparison of the two sensor responses to the T1, T4, and T5 stages; same colour coding and fittings of (**a**).

After enough time from T3, the responses of ST25 and STN sensors decreased progressively and became 15.5%, 21% and 17.8%, 24% smaller after one year (T4) and three years (T5) of follow up, respectively (Figure 5a). All the T4 and T5 responses were significantly smaller than the T1 ones, consistently with the fact that, luckily, none of the recruited patients had relapses (Figure 5b). Indeed, the surgical removal of the tumoral mass had completely removed the cancer, as proved by the clinical lack of relapses, and consequently the cell VOCs dumped in the blood stream. Accordingly, the T4 and T5 responses are comparable with the ones obtained for healthy people in the previous studies [21,30,44].

**Figure 6:**
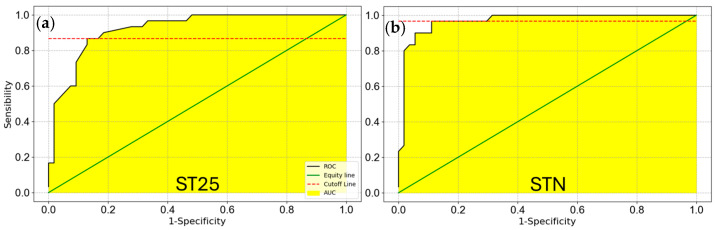
T1 vs. T5 ROC curves. The sensor capability to discriminate between T1 and T5 samples. (**a**), ROC curve of ST25 sensor (AUC = 0.93); (**b**) ROC curve of STN sensor (AUC = 0.97).

The average ST25 responses to T1 and T5 samples were 1.49±0.04, (CI = 1.4–1.5) and 1.21±0.01, (CI = 1.18–1.2), respectively, both appearing normally distributed. To statistically highlight a possible difference between T1 and T5 sample means, the Welch’s *t* test was performed, being a significance *t*-test for unpaired normally distributed data with different variances, as highlighted by Levene’s test [68] *p*-value of 1.3 × 10^−7^ (*p* << 0.05). The Welch’s *t*-test, with a *p*-value of 8.6 × 10^−7^ (*p* << 0.05), forced the null hypothesis (mean (T1) = mean (T5)) rejection, demonstrating that T1 and T5 samples owned to two well separated distributions (thesis confirmed also by the completely non-overlapping CIs). Moreover, to quantify the surgical treatment effect, the Cohen’s d effect size was computed [69,70], resulting in 1.82 (>0.8), highlighting a large effect size. Similar results were obtained for the STN sensor: the T1 and T5 averages were 1.65±0.05 and 1.24±0.01 respectively; Levene’s test highlighted a difference in the sample variances with a *p*-value of 1.6 × 10^−6^ (*p* << 0.05); Welch’s *t* test, with a *p*-value of 3.2 × 10^−8^ (*p* << 0.05) leads to the rejection of the null hypothesis, highlighting that T1 and T5 represent two different distributions. Finally, the Cohen’s d resulted to be 2.24 (>0.8), showing a huge effect size between T1 and T5 samples. The sensor capability in discriminating between the T1 and T5 samples was further evaluated by means of the Receiver Operating Characteristic (ROC) curves [71,72,73] (for each sensor; Figure 6), giving an Area Under Curve (AUC) higher than 0.9 for both sensors (where an AUC of 1 indicate a perfect test, while an AUC of 0.5 a test giving a random result).

**Figure 7:**
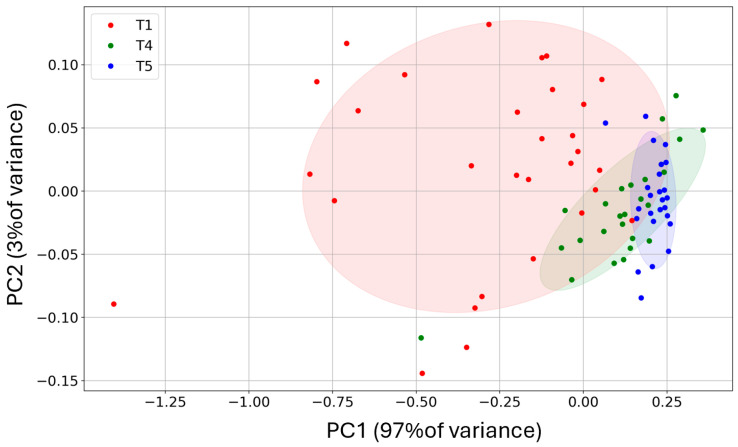
Principal component analysis of the T1, T4, and T5 sensor responses. The ST25 + 1% Au and STN sensor responses were processed to obtain a qualitative classification of the samples: T1 (red), T4 (green), and T5 (blue) points; the light red, light green and light blue regions are the corresponding ellipses of confidence.

The discriminating power between T1, T4, and T5 of the two sensors was further qualitatively evaluated with the PCA [74,75], useful technique to remove possible redundancy and noise in a dataset without losing data information (variances). Indeed, the data plotted in the PC’s reference frame appear completely without any correlation (covariances = 0) and with the variances maximized along the axis. The two eigenvectors of the covariance matrix (PC1 and PC2), computed from the two sensor responses, were drawn one vs. the other to construct the two-dimensional score (or dispersion) plots. The fraction of variance (in percent) of the datasets projected on the PCs were 97% for PC1 and 3% for PC2, giving a distribution of T1 samples (red) well discriminated against to the T4 (green) and T5 (blue) ones (Figure 7); the T4 and T5 samples were well superimposed, indicating that at stage 4 the patient was substantially healed.

**Figure 8:**
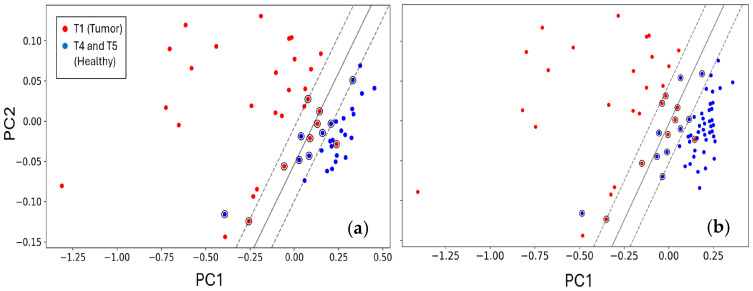
Data discrimination by SVM. (**a**) The responses of ST25 + 1%Au and STN sensors relative to the T1 (red) and T4 (blue) periods; (**b**) their responses to T1 (red), and T4 and T5 (blue) periods. The solid line marks the separation between healthy subjects and the cancer affected patients, while the dashed lines represent the margins.

The data was more quantitatively classified by using the supervised learning method known as the support vector machine (SVM) [76,77]. SVM is used here to detect a possible linear cutoff among data by maximizing their classification through the use of the support vectors [77]. Here the kernel used for the decision function was linear [78], to find out the best line discriminating T1 from T4 (Figure 8a), and T1 from T4–T5 (Figure 8b). It was then necessary to set the regularization parameter c, controlling the tradeoff between minimizing the training error and the testing error; the latter represents the capability of the algorithm to successfully classify new data, i.e., not used for training. A small value of c (1–10) leads to a large window (margin) in discriminating between the two classes considered here (T1 and T4 and T1 and T4–T5) and therefore it is prone to misclassify the training data, therefore c was set to 1000.

### 3.2. Three-Years Follow Up: Gender Analysis

**Figure 9:**
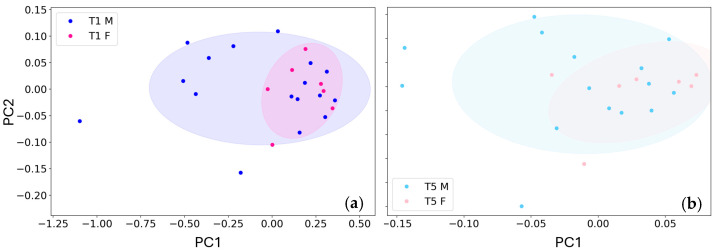
Principal component analysis comparing the T1 and T5 sorted by gender. (**a**) Sensor responses of male (T1 M) and female (T1 F) tumor affected subjects and (**b**), healthy ones (T5 M and T5 F); the corresponding confidence ellipses are drawn with lighter colors in both panels.

Of the 30 patients considered here, just 24 provided T1, T4 and T5 samples, since six patients provided the T1 sample only, but they could not return for the annual control, when the T4 and T5 were collected, therefore we narrowed down the statistical analysis to 24 patients only to assess the possible gender dependency of the sensor responses. In order to remove any correlation (information redundancy) between the two sensor outcomes [79], and to obtain a dataset representing them concurrently, the dataset was at first processed with PCA and the data were then represented in the PC1 vs. PC2 reference frame (Figure 9) to compare T1 and T5 male vs. female subjects separately.

The data points distributions related to male and female were graphically superimposed (i.e., undistinguishable) in both T1 and T5 plots. The ST25 and STN sensor responses were then processed with PCA and the PC1 projections were employed to perform the statistical significance tests, by using an alpha (significance parameter) of 0.05. In order to assess the data normality, the Shapiro-Wilk test (SWT) [80,81,82] was performed separately on the four datasets T1 male, T1 female, T5 male, and T5 female. The SWT results showed that T1 and T5 female datasets are normally distributed (*p*-values of 0.51 and 0.22), while the others are randomly distributed (*p*-values of 0.03 and 0.05). Therefore, all the datasets were treated as non-normally distributed and the statistical significance of T1 male vs. female and T5 male vs. female datasets were assessed through the Mann-Whitney test (non-parametric test for unpaired samples) [83,84]. The two (male and female) T1 datasets and the two T5 ones resulted included in the same distributions, with *p*-values of 0.22 and 0.23, respectively. Given the lack of statistical differences between the two groups, we then included in the above statistical analysis all the male and female subjects that were collected in previous work [21].

### 3.3. Global Gender Statistical Analysis

**Figure 10:**
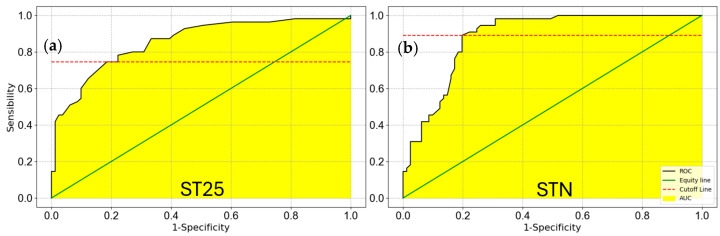
T1 vs. T5 ROC curves. The sensor capability to discriminate between T1 and T5 samples. (**a**), ROC curve of ST25 sensor (AUC = 0.88); (**b**) ROC curve of STN sensor (AUC = 0.90).

Since the T1 samples arise from CRC patients and the T4 and T5 samples from healed patients, it is reasonable that the former are similar to the previously collected tumoral samples, while the latter to healthy ones, as shown by the ROC curves (Figure 10). Therefore the data collected from the aforementioned feasibility previous studies were unified with the ones of this study.

**Figure 11:**
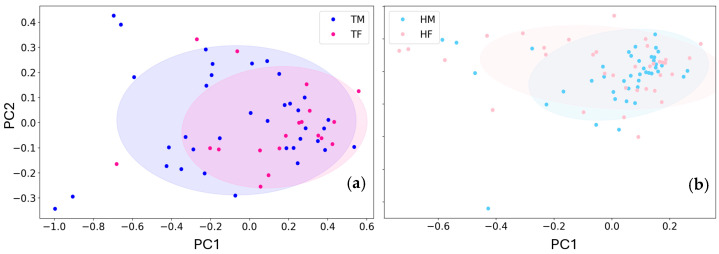
Global health and tumor affected subject responses sorted by gender. (**a**) Sensor responses of male (TM) and female (TF) tumor affected samples and (**b**), healthy ones (HM and HF); the corresponding confidence ellipses are drawn with lighter colors in both panels.

Therefore, to increase the sample size regarding the gender, we included these previously collected data to the above statistics, giving 36 and 19 tumor affected male and female samples, respectively, and 46 and 35 healthy male and female ones, respectively. The responses of the two sensors considered to these 136 samples were processed, at first by using PCA method, in order to represent their concurrent responses and to remove any correlation between them. The two-dimensional PC1 vs. PC2 score plots sorted for male and female healthy subjects and of tumor affected ones are shown in Figure 11a and Figure 11b respectively.

Again, the data points distributions related to male and female were undistinguishable (as in Figure 9). The statistical significance tests were performed by using the PC1 projections as above, by using an alpha of 0.05. The SWT was performed separately on the four datasets TM (tumor affected male samples), TF (tumor affected female samples), HM (healthy male samples), and HF (female healthy samples). The SWT results showed that the TF dataset is normally distributed (*p*-value of 0.13), while all the others (TM, HM and HF) are randomly distributed (*p*-values << 0.05). Therefore, all the datasets were treated as non-normally distributed, and the statistical significance of TM vs. TF and HM vs. HF were assessed through the Mann-Whitney test (non-parametric test for unpaired samples). In both cases the *p*-values were 0.28 and 0.61, therefore, being much larger than 0.05, they are included in the same distributions. Again, no statistical differences were found between male and female. This confirm a previous study indicating relatively small gender differences in screening uptake, route to diagnosis, cancer staging at diagnosis and survival, suggesting that the higher CRC mortality in men appears to be a result of endogenous and/or exogenous pre-diagnosis factors leading to higher incidence rates [85].

## 4. Conclusions

SCENT B2 selected sensors (ST25 and STN) resulted to be suitable to reliably monitor the patient’s health status in a follow-up protocol up to 3-years. The PCA and SVM plots showed a good discrimination between tumoral (T1), and healthy samples (T4 and T5), from a qualitative and quantitative point of view, respectively. The statistical significance tests confirmed what was inferred by PCA and SVM analysis, highlighting a huge effect size (2.24, much larger than 0.8) for T1 vs. T5. As confirmed in a double-blind approach by the clinical follow-up, no relapses were detected. Concerning the gender dependency of the sensor responses (through the Mann-Whitney non-parametric test), it can be concluded that the patient’s gender does not affect the sensor responses (*p*-values > 0.05). The brilliant performances of SCENT B2 sensors in discriminating tumoral blood from the healthy one, not only between tumor affected and healthy subjects, but also in a complex follow-up protocol, make it a future opportunity to improve the tumor screening protocols and the patient’s follow-up reliability. Nevertheless, to validate this device and to adopt it in clinical practice it is necessary to:

- enlarge the patient number in order to make the results more robust and reliable;
- further improve the SCENT B2 device to make it stand alone (currently in development);
- sensor technology improvements in order to better detect the CRC stages and to extend the use of this device for other tumor types;
- test the device in the case of gastrointestinal conditions such as Crohn’s disease, irritable bowel syndrome, and ulcerative colitis. Indeed, besides detecting the VOCs produced by the cancer cells, the sensors could detect also the VOCs produced by the local bowel inflammation triggered by the cancer itself. Therefore, in the case of non-cancerous inflammation, the bowel cells (and the ones that may be recruited in the inflamed region) could dump in the blood stream some VOCs that could be detected by the chemoresistive sensors as well, possibly giving a false positive.

Finally, this device is planned to be used in the future to detect other highly vascularized cancer types that dump VOCs in human body fluids that can be collected in deciliter volumes (as blood, urine, feces, edemas, etc.), as the vascular tumors.

## 5. Patents

The Scent B1 device is patented in Italy with patent number: 102015000057717.

## Figures and Tables

**Table 1:** CRC incidence and death percentage in the Italian regions.

Italian Region	Population × 10^6^	Incidence	Death	Inc. %	Death %
Lombardy	10	65,000	19,000	0.65	0.19
Latium	5.7	34,000	10,000	0.60	0.18
Campania	5.7	31,000	9000	0.54	0.16
Veneto	4.9	31,000	8500	0.63	0.17
Emilia Romagna	4.4	30,000	8000	0.68	0.18
Piedmont	4.3	29,000	7500	0.67	0.17
Sicily	5	27,000	8000	0.54	0.16
Tuscany	3.7	27,000	7000	0.73	0.19
Apulia	4	23,000	6500	0.58	0.16
Calabria	1.9	10,000	3500	0.53	0.18
Liguria	1.5	9000	3000	0.60	0.20
Friuli	1.2	8000	2500	0.67	0.21
Sardinia	1.6	8000	2500	0.50	0.16
Marches	1.5	7000	2500	0.47	0.17
Abruzzo	1.3	6000	2000	0.46	0.15
Trentino	1.1	5000	1500	0.45	0.14
Umbria	0.88	4000	1500	0.45	0.17
Basilicata	0.54	2000	1000	0.37	0.19
Molise	0.3	1500	600	0.50	0.20
Aosta Valley	0.125	700	250	0.56	0.20

**Table 2:** Recruited Patients.

Patient Feature	Type	N. Patients	Percentage/Range
SEX	Male	21	64%
	Female	12	36%
AVERAGE AGE	Male/Female	69	47–87
BMI	>30	9	29%
	<30	22	71%
Tumor Localization	Ascending Colon	20	61%
	Transverse Colon	4	12%
	Descending Colon	3	9%
	Sigma	3	9%
	Rectum	3	9%
Tumor Stage	I	3	10%
	II	14	45%
	III	13	42%
	IV	1	3%

## Data Availability

Data is unavailable due to privacy or ethical restrictions.
